# Chemical and Functional Consistency of Condensed Tannins from *Anadenanthera macrocarpa* at Different Collection Sites

**DOI:** 10.1021/acsomega.5c09054

**Published:** 2026-02-17

**Authors:** Kamilla V. R. de A. Silva, José Erick Galindo Gomes, Keila Aparecida Moreira, Rodrigo José da Silva Lima, Pedro L. Guzzo, Dulciene Karla de Andrade Silva, E. Padrón Hernández

**Affiliations:** † Universidade Federal de Campina Grande, Unidade Acadêmica de física, Rua Aprígio Veloso, 58109-970 Campina Grande, PB, Brazil; ‡ 667896 Universidade Federal do Agreste de Pernambuco, Laboratório Caatinga BioAtiva- PPGCAP, Nutrição animal e Biocompostos, Av. Bom Pastor, s/n, 55292-270 Garanhuns, PE, Brazil; § 154624 Universidade Federal de Campina Grande, Laboratório de Espectroscopia Fotoacústica, Rua Aprígio Veloso, 58109-970 Campina Grande, PB, Brazil; ∥ University of Copenhagen, Department of Chemistry, Universitetsparken 5, 2100 Copenhagen Ø, Denmark; ⊥ 28116 Universidade Federal de Pernambuco, Departamento de Engenharia de Minas, Av. Prof. Moraes Rego, 1235, 50670-901 Recife, PE, Brazil; # 28116 Universidade Federal de Pernambuco, Departamento de Física, Av. Jorn. Aníbal Fernandes, s/n, 50740-540 Recife, PE, Brazil; ∇ 28116 Universidade Federal de Pernambuco, Pós-graduação em Ciência dos Materiais, Av. Jorn. Aníbal Fernandes, s/n, 50740-540 Recife, PE, Brazil

## Abstract

Condensed tannins (CTs) are versatile polyphenolic compounds with significant industrial potential attributable to their antimicrobial, antioxidant, and adhesive properties. In this context, the study of species with a high tannin content and large-scale production capacity is crucial for expanding commercial applications. Among tannin-rich species, *Anadenanthera macrocarpa* (Benth.) stands out for its high yield and suitability for diverse industrial uses. However, understanding the influence of edaphoclimatic conditions on the chemical stability of CTs in this species is essential to ensure the consistency and quality of the extracted tannin. In this sense, this study analyzed CT extracts from *A. macrocarpa* that were collected from three distinct locations with varied soil and climate conditions. Five plants in consistent vegetative phenological stages were selected from each site for representative sampling. The extracts were characterized by using spectroscopic techniques, including NMR, HSQC, EPR, FTIR, and UV–vis, alongside thermogravimetric analysis (TGA). Chemical characterizations indicate that samples from different locations exhibit a similar overall chemical profile within the sensitivity of the techniques employed, suggesting resilience to environmental variations and supporting their potential reliability for large-scale extraction. Additionally, antimicrobial assays were conducted against bacterial strains, *Staphylococcus aureus* and *methicillin-resistant* *S. aureus* (MRSA), at different concentrations. The highest concentration tested (1000 μg/mL) proved to be the most effective, exhibiting an antimicrobial activity of 74.81 ± 1.42%, revealing significant inhibitory effects. The antioxidant activity was also evaluated, reinforcing their applicability in the industrial and biomedical fields. Our results indicate that the condensed tannins extracted from *A. macrocarpa* exhibit strong potential for large-scale production, in addition to possessing antioxidant and antimicrobial activity of interest for various biomedical applications.

## Graphical abstract

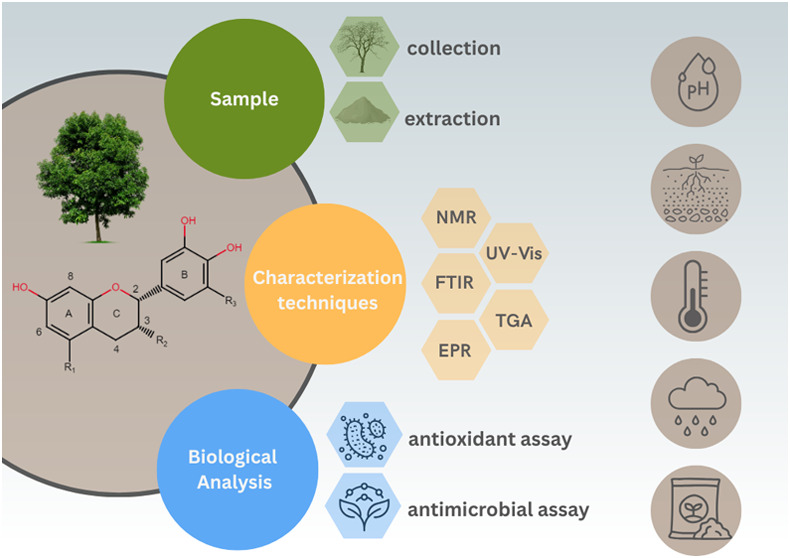

## 1 Introduction

Condensed tannins (CT) are polyphenolic compounds found in various plant species, produced in response to environmental and biological stress factors.^1,2^ Because of their expressive antioxidant and antimicrobial activities, they have been investigated for applications in different technological areas, including pharmaceutical, cosmetic, food, and biotechnology sectors.^3–6^ In orthopedics, tannin can be used as a coating on metallic surfaces, making the implant-bone interface more favorable for osseointegration due to the presence of hydroxyl groups with high electron-donating capacity, which are capable of neutralizing oxygen free radical.^7,8^ Their antimicrobial activity was evaluated against different strains of Gram-positive and Gram-negative bacteria, with observed bacteriostatic potential.^9,10^ In addition to biomedical uses, condensed tannins are applied as natural adhesives and in leather tanning processes.^11–14^ The versatility of this material has motivated the investigation of condensed tannins as promising agents for innovative solutions.^11,15–17^

Also known as proanthocyanidins-prodelfins, CTs present a highly diverse chemical structure. Their molecular configuration involves units of flavan-3-ol, gallocatechin, and epigallocatechin.^1,18^ Its monomeric units, composed of (+)-catechins, (−)-epicatechins, are linked by carbon–carbon bonds, resulting in oligomeric and polymeric structures,^1,2,19,20^ as shown in Figure 1. Such structural complexity grants CTs a remarkable ability to form complexes with proteins, polysaccharides, and other compounds.^19,21^ However, structural conformation is significantly influenced by variables such as the plant growth area, botanical source, and environmental conditions affecting the sample, which is reflected in the wide range of molecules with functional potential in different applications. Nevertheless, this variability can represent a critical challenge, since chemical stability and compositional consistency are essential requirements for large-scale applications.

**Figure 1:**
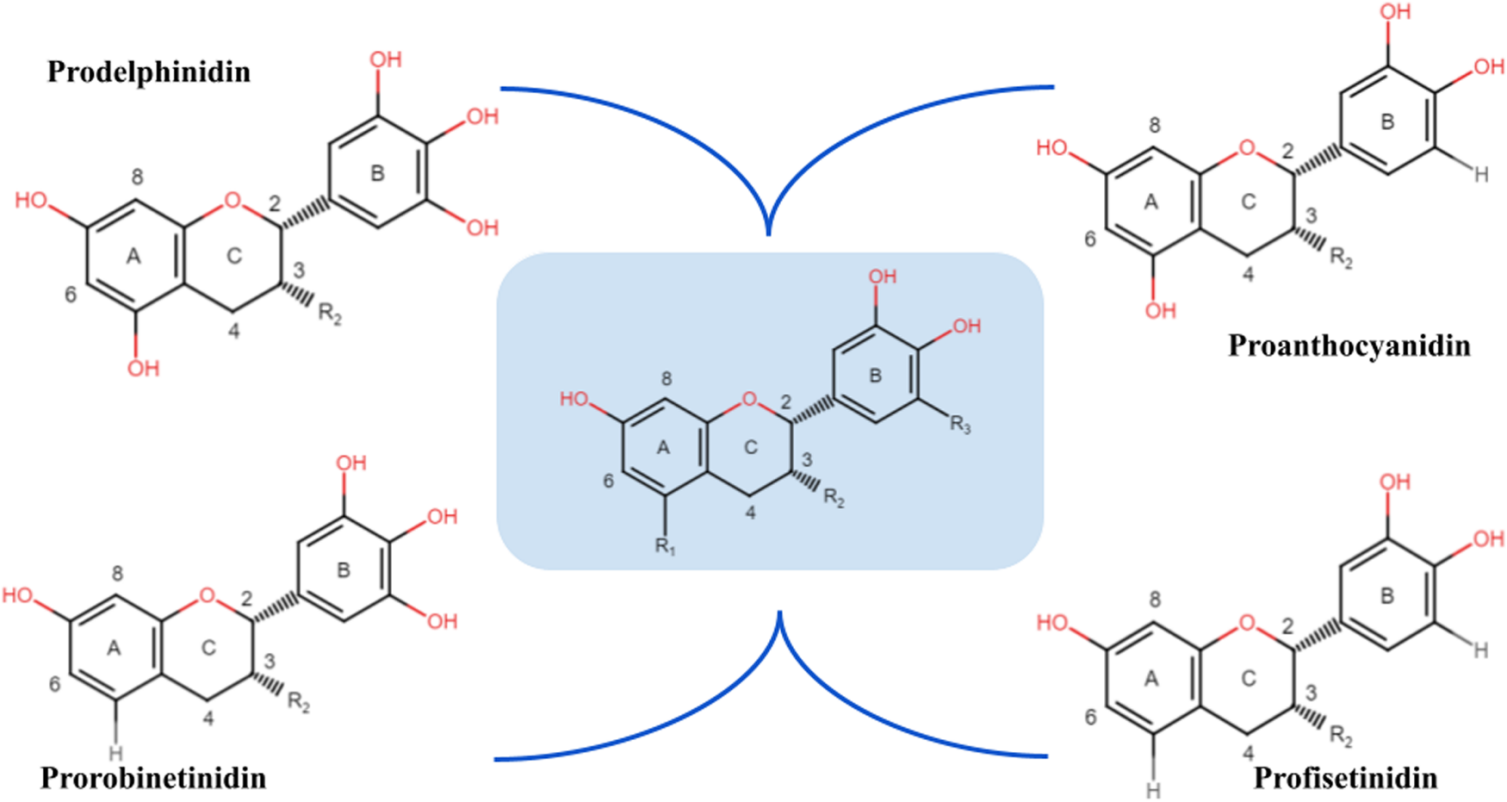
Chemical structure and nomenclature of condensed tannin monomers.

Among tannin-rich species, *Anadenanthera macrocarpa* (red angico) stands out due to its high tannin content and adaptability to diverse edaphoclimatic conditions.^22,23^ Native to the Caatinga and Cerrado biomes, this species shows fast to moderate growth, reaching 5–6 m within the first two years and up to 20 m in adulthood, with average productivity between 25.55 and 39.98 m^3^ ha^–1^ year.^24–26^ It presents significant potential for reforestation, recovery of degraded areas, and production of high-quality wood.^27^ The genus *Anadenanthera* is distinguished by its high content of condensed tannins, especially in the bark, ranging from 118.9 g kg^–1^ dry matter (DM) in *A. colubrina* to 125.7 g kg^–1^ DM in *A. macrocarpa*.^28,29^ These bioactive compounds support multiple industrial uses, including leather processing, adhesive formulations, biopolymers, and chemical and pharmaceutical applications.^30–32^

Despite their economic and environmental value, the effects of environmental variability on the structure and functionality of tannins are still poorly understood. Factors such as soil composition, rainfall, and pests can influence the stability and reactivity of these compounds. Understanding these interactions can improve the sustainable cultivation of this species, support ecosystem restoration, carbon sequestration, and income generation in semiarid regions, positioning *A. macrocarpa* as a promising and sustainable natural source of tannins.

Given these considerations, this study investigates the chemical structure of condensed tannins extracted from *A. macrocarpa* collected in three regions with contrasting edaphoclimatic conditions. To this, we employed complementary spectroscopic and thermal techniques, including UV–vis spectroscopy, Fourier transform infrared spectroscopy (FTIR) through the ATR method, nuclear magnetic resonance (NMR), heteronuclear single quantum coherence (HSQC), electron paramagnetic resonance (EPR), and TGA, to evaluate whether environmental variation influences structural features and thermal stability. Additionally, its antimicrobial activity will be evaluated against strains of methicillin-resistant *S. aureus* (MRSA) and *S. aureus*, as well as its antioxidant activity, as depicted in Figure 2.

**Figure 2:**
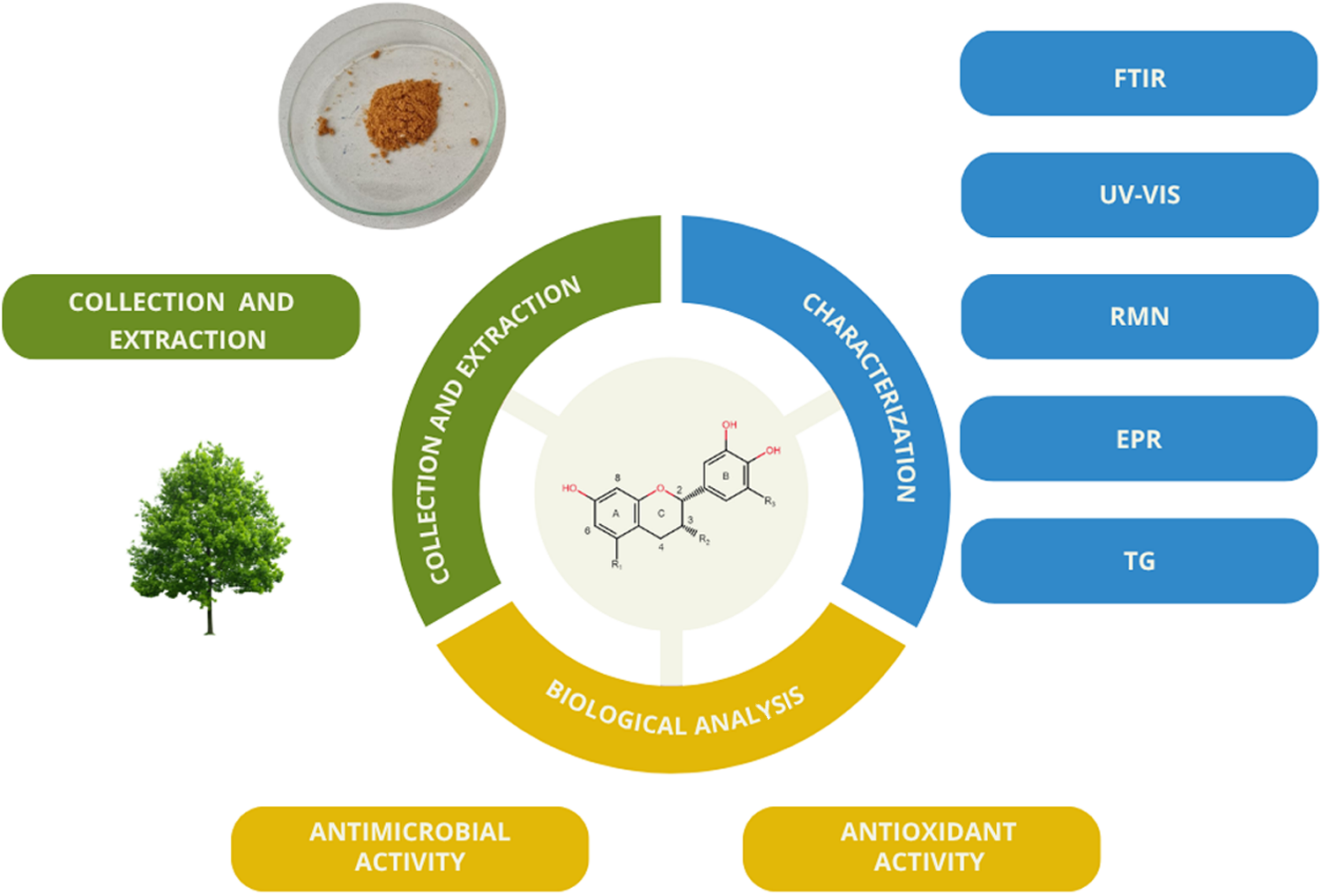
Steps developed in this work are summarized in a summarized form.

By doing so, we seek to provide information about the stability of tannins under diverse environmental conditions, reinforcing their potential as reliable bioactive materials for industrial applications and large-scale extraction. Altogether, this comparative approach allows us to examine the extent to which condensed tannins from *A. macrocarpa* remain chemically consistent across the regions.

## 2 Materials and Methods

### 2.1 Collection and Extraction

Condensed tannin extracts (TC) from *A. macrocarpa* were used, and the species was registered in SisGen: AAE7054 in accordance with the provisions of Law n.13.123/2015 and its regulations. To ensure the representativeness of the samples, five plants for each of the three locations were chosen with similar characteristics in terms of vegetative phenological stage, with the presence of fully expanded leaves and the absence of senescent leaves, flowers, and fruits.^23^

The collected samples were dried in an oven at 40 °C until a constant weight and ground in a Wiley mill to a particle size of 1 mm. The determination of free condensed tannins (CT) and the protein-bound and fiber-bound fractions followed the methodology of Terril et al.^33^ Extraction was performed using an acetone/water solution (70:30, v/v) and concentrated ethyl ether, followed by purification and molecular-size fractionation on a Sephadex LH-20 column. Protein-bound and fiber-bound fractions were extracted using sodium dodecyl sulfate (SDS) and 2-mercaptoethanol. Quantification of all fractions was carried out using a butanol–HCl solution (95:5, v/v), according to Porter et al.,^34^ based on the formation of colorimetric anthocyanidins measurable by spectrophotometry. According to the methodology used, the yield was 125.7 g of tannins obtained from 1 kg of dry mass of red angico bark.

The samples are labeled as TAVAL, TAVPE, and TAVPB, with variations corresponding to the collection location.

### 2.2 Meteorological and Soil Data

Meteorological data, including information on average precipitation and temperature, as well as details on soil conditions and composition, were obtained from a previous study conducted by Souza et al.^23^ Tables 1 and 2 summarize these parameters, as reported in the previous study. This prior study analyzed the same samples used in the present work, ensuring representativeness and standardization of the environmental data. These data were integrated into this study to contextualize the environmental differences to which the samples were exposed.

**Table 1:** Meteorological Data for the Sampling Sites during the Collection Period (Adapted from Souza *et al*., Rev. Ciênc. Agron. 51 (3), 2020. Licensed under CC BY 4.0.^23^)

sample	coordinate	altitude (m)	year rainfall average (mm)	minimum average air temperatures (°c)	average air temperatures (°c)	maximum air temperatures (°c)
TAVAL	37°57′18″W and 9°23′42″S	256	33	27	29	32
TAVPE	36°57′5″W and 8°19′00″S	663	105	19	24	27
TAVPB	37°16′30″W and 7°01′30″S	242	11	29	30	32

**Table 2:** Soil Composition Data for the Sampling Sites (Adapted from Souza *et al*., Rev. Ciênc. Agron. 51 (3), 2020. Licensed under CC BY 4.0.^23^)

sample	coordinate	pH	organic matter (%)	sand (g.kg^1^)	silt (g.kg^1^)	clay (g. kg^‑1^)
TAVAL	37°57′18″W and 9°23′42″S	5.7	1.6	779.47	178.53	42.00
TAVPE	36°57′5″W and 8°19′00″ S	6.1	5.2	572.47	337.53	90.00
TAVPB	37°16′30″ W and 7°01′30″ S	6.4	2.1	726.96	203.04	70.00

### 2.3 Nuclear Magnetic Resonance (NMR)

Liquid-state ^1^H, ^13^C, and HSQC nuclear magnetic resonance (NMR) spectra were recorded at 27 °C using a 500 MHz Ultrashield Plus 500 spectrometer and a 125 MHz Bruker instrument (acquisition time of 4 h each). Chemical shifts (δ) are reported in parts per million (ppm), and coupling constants (*J*) are denoted in Hertz (Hz). The samples analyzed by NMR were dissolved in DMSO-d6 at room temperature.

### 2.4 UV–Vis Spectroscopy

The UV–vis spectroscopy is based on the amount of light a substance absorbs as a function of wavelength. This absorption occurs because the molecules composing this material have electrons that can be excited by absorbing a certain amount of energy. This technique is widely used in condensed tannins to identify the existing chromophore groups in this material. The UV–vis measurements were conducted at room temperature using a Cary 5000 UV–visNIR spectrophotometer (Agilent Technology), covering the range of 200–3300 nm. Prior to measurement, the samples were appropriately diluted in deionized water and transferred to quartz cuvettes. A standard curve was established by using a sample of deionized water in a quartz cuvette. The measurements were performed in duplicate to ensure reproducibility and accuracy. Additionally, all measurements were conducted under controlled environmental conditions to minimize potential variability.

### 2.5 Fourier Transform Infrared Spectroscopy (FTIR)

FTIR spectroscopy is a widely used analytical method for determining chemical compounds, providing a fingerprint for different types of tannins. The vibrational absorption spectra were obtained using a Bruker Vertex70 spectrometer, employing the attenuated total reflection (ATR) technique with a Pike Miracle accessory equipped with a zinc selenide crystal (ZnSe). Spectra were acquired in the range of 4000 to 600 cm^–1^, with a resolution of 4cm^–1^ and 32 scans. Samples were directly deposited onto the ATR crystal and pressed to ensure good contact.

### 2.6 Thermogravimetric Analysis (TGA)

In our study, thermogravimetric analysis (TGA) was employed to gain insight into the biostability of condensed tannin. This method allows for the observation of mass variation attributed to interactions with the atmosphere, vaporization, and decomposition. The measurements were performed with the aim of evaluating atomic interactions and connectivity within the molecules that make up the condensed tannin, in order measurements were conducted under an air atmosphere, with heating initiated from room temperature up to 700 °C at a rate of 20 °C/min.

### 2.7 Electron Paramagnetic Resonance (EPR)

The electron paramagnetic resonance (EPR) measurements were performed on a Bruker EMX 10+ spectrometer operating at X-band frequencies with a cylindrical cavity. At room temperature, with a modulation of amplitude 2.0 G, frequency 9.7503 GHz, microwave power 6.33 mW, and conversion time of 25 ms.

### 2.8 Antimicrobial Activity

Antimicrobial activity was assessed using the turbidity method described by Wu et al.^35^ with some modifications. The bacterial strains *S. aureus* and methicillin-resistant *S. aureus* PE 86 (MRSA) (provided by Fundação Oswaldo Cruz, Fiocruz) were used. Sterile 96-well flat-bottom polystyrene microplates were used for the assay. The CT samples were diluted to achieve concentrations of 1000, 500, 250, 125, and 62.5 μg/mL. A 50 μL aliquot of each sample was added to 45 μL of Mueller-Hinton broth (MHB) and 5 μL of the bacterial suspension. A blank was prepared containing 50 μL of the plant extract at different concentrations and 50 μL of MHB broth. The positive control contained only Mueller-Hinton broth (MHB) and the bacterial suspension, while the negative control included chloramphenicol at a concentration of 300 μg/mL. After incubating the plates at 37 °C for 24 h, the absorbance was measured using a microplate reader at 620 nm.

#### 2.8.1 Antioxidant Activity

##### 2.8.1.1 Radical Scavenging Activity of ABTS^+•^ and DPPH

The free radical scavenging activity of purified tannin was determined with the ABTS^+•^ radical cation, generated from 2,2-azinobis-3-ethylbenzothiazoline-6-sulfonic acid (ABTS) according to Re et al.^36^ with modifications. For ABTS^+•^ radical formation, potassium persulfate and ABTS were prepared at final concentrations of 2.45 and 7 mM, respectively, and incubated in the dark for 16 h at room temperature. The absorbance of the radical solution was adjusted to 0.70 ± 0.02 at 734 nm, with a spectrophotometer, by dilution in 100 mM phosphate-buffered saline (PBS), pH 7.4. For the reaction, 50 μL of the samples were mixed with 950 μL of the ABTS^+•^ radical solution. The assays were incubated at 30 °C for six min and read at 734 nm, in triplicate. The standard curve was obtained with Trolox (20–800 μM) as the standard antioxidant substance. The antioxidant activity (%) was calculated in relation to the radical scavenging activity according to the following equation: Antioxidant Activity (%) = [(*A*_control_ – *A*_sample_)/*A*_control_] × 100, where *A*_control_ represents the initial absorbance of ABTS and A_sample_ the absorbance after the addition of the respective sample. Another free radical used to evaluate the potential antioxidant activity was 2,2-diphenyl-1-picrylhydrazyl (DPPH). The evaluation of DPPH scavenging activity was performed according to the methodology described by Brand-Williams et al.^37^ with some modifications. The DPPH radical (0.2 mM) was prepared in ethanol. For the reaction, 750 μL of the sample was mixed with 750 μL of the solution with the DPPH radical. The reaction occurred for 30 min protected from light, and the reading was performed in a spectrophotometer at 517 nm. The standard curve was prepared with ascorbic acid (1–30 μM) as the standard antioxidant. The antioxidant activity (%) was calculated following the same formula as the ABTS methodology.

##### 2.8.1.2 Metal Ion Chelating Activity

The copper (Cu^2+^) chelating activity was determined according to Sánchez-Vioque et al.^38^ and modified. For the reaction, 0.5 mL of the sample was mixed with 2 mL of 50 mM sodium acetate buffer, pH 6.0, and 50 μL of 5 mM CuSO_4_. After 30 min of incubation at room temperature, 50 μL of 4 mM pyrocatechol violet (PV) was added. The reaction mixture was homogenized, and the reaction continued for another 30 min before measurement at 620 nm. Deionized water was used as a negative control, replacing the sample, and a 0.045% EDTA solution was used as a standard chelating substance. The percentage of inhibition of the formation of the PV–Cu^2+^ complex was calculated as follows: [(*A*_0_–*A*_1_)/*A*_0_]×100, where *A*_0_ corresponds to the absorbance of the negative control, and *A*_1_ to the absorbance of the samples.

Iron chelating activity (Fe^2+^) was determined according to the methodology proposed by Sánchez-Vioque et al.^38^ For the reaction, 0.5 mL of the sample was mixed with 2 mL of 100 mM sodium acetate buffer, pH 4.9, and 50 μL of 2 mM iron­(II) chloride. After 30 min of incubation at room temperature, 200 μL of 5 mM ferrozine was added. The reaction mixture was homogenized, and the reaction continued for another 30 min before measurement at 562 nm. Deionized water was used as a negative control, replacing the sample, and a 0.045% EDTA solution was used as a standard chelating substance. The percentage of inhibition of the formation of the ferrozine-Fe^2+^ complex was calculated following the same formula as the methodology used for copper chelating activity.

### 2.9 Statistical Analyses

Statistical analysis was performed using the R statistical software (R CORE TEAM, 2024). For antimicrobial activity, one-way ANOVA followed by Tukey’s test was applied to assess statistical differences (*p* < 0.05) among the tested concentrations, as well as between the collection sites of the plant material and the microorganism. For antioxidant activities, one-way ANOVA followed by Tukey’s test was applied to assess statistical differences (*p* < 0.05) among the tested concentrations and between the collection sites of the plant material.

## 3 Results and Discussion

### 3.1 Nuclear Magnetic Resonance (NMR)

The ^13^C spectra of the samples revealed a consistent profile across different environmental conditions, with no significant deviations in the chemical shifts observed, Figure 3. A peak at 144.4 ppm was identified, corresponding to the B-ring carbons, specifically of procyanidin monomer units (C–3′,C–4′), and of prodelphinidin units (C–3′, C–5′), which are characteristic of catechin and gallocatechin substructures, respectively.^39–41^ These signals suggest that the CTs of this species are composed of a mixture of procyanidin- and prodelphinidin-type flavan-3-ol units, consistent with common structures found in hardwood species. Studies by Zhang et al.^42^ show that this peak can be much smaller if it is considered an aminated tannin, which may indicate that some hydroxyls of the B ring may have been replaced by–NH_2_.

**Figure 3:**
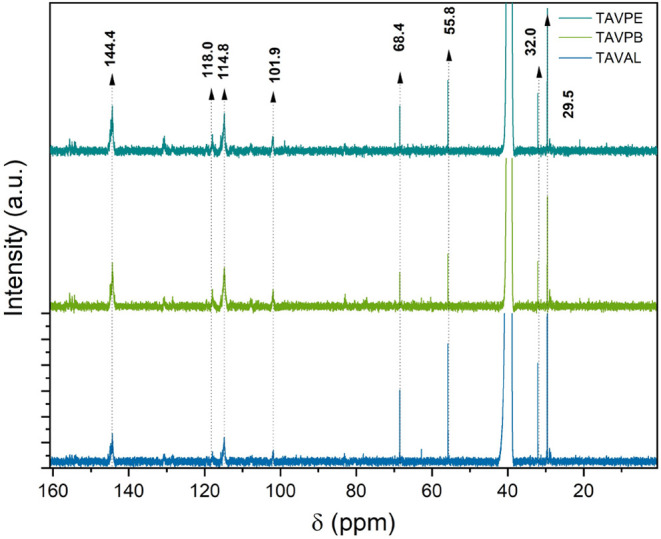
^13^C NMR spectrum of the condensed tannin from *A. macrocarpa* in DMSO-*d*_6_.

Additional peaks observed between 101.9 and 118.0 ppm were attributed to C–4a and C–8 carbons, characteristic of procyanidin and prodelphinidin monomeric units.^39,43^ Specifically, the peaks 114.8 and 118.0 ppm can also be attributed to carbons C2′, C5′, and C6′ of the B ring, supporting the presence of catechol-type (3′,4′-dihydroxy) substitution in procyanidins, and possibly pyrogallol-type (3′,4′,5′-trihydroxy) in prodelphinidins.^2,41,44^ This substitution pattern is diagnostic of the flavonoid origin and is central to their antioxidant and metal-chelating capacity. A chemical shift at 68.4 ppm was attributed to C–3′ in cis and trans configurations within terminal units, while multiple signals in the range of 29.5–32.0 ppm were associated with C–4 carbons in cis and trans configurations at the terminal positions and offer indirect evidence of interflavan bonds.^45^ These observations align with those from prior studies and confirm the structural consistency of the condensed tannins across different samples.

In Figure 4, peaks around 8.4–9.1 ppm were identified, and according to studies carried out by Duval et al.^2^ for the condensed tannin of *A. catechu*, they can be attributed to a phenolic OH region. In that same work, it was observed that when adding D_2_O, the H NMR signal undergoes a strong decrease.

**Figure 4:**
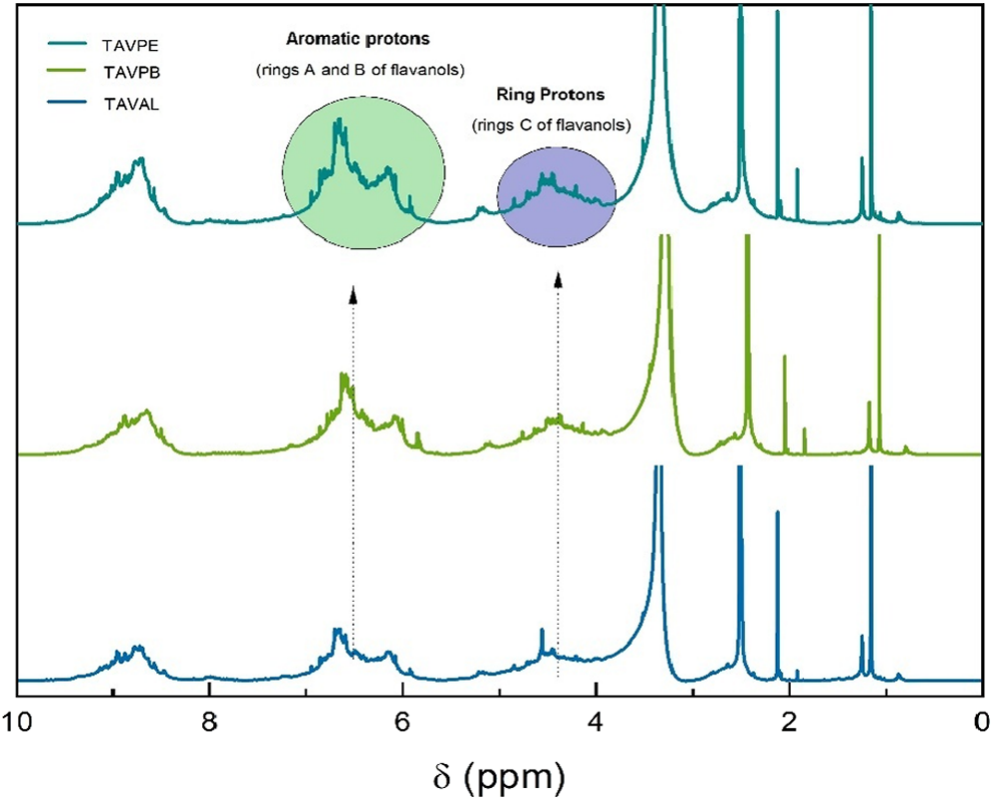
^1^H NMR spectrum of the condensed tannin from *A. macrocarpa* in DMSO-*d*_6_.

Additionally, a set of signals typical of aromatic regions between 5.8 and 6.9 ppm are observed and are signed by the A and B rings of flavonoids. This region supports the presence of resorcinol and catechol substitution patterns, which are common in procyanidin and prodelphinidin units. In the characteristic shift range for the methine and methylene protons of the dihydropyran moiety (4.7–2.8 ppm), the signals can be associated with the C ring of the flavan-3-ol units of condensed tannins. These signs may indicate the predominant presence of proanthocyanidin oligomers of this fraction.^46^

The absence of significant cross-peaks of non-CT components from ^1^H and ^13^C NMR graphical data indicates the purity of the condensed tannins, which corroborates the other analyses carried out in the present work. From the 2D NMR data, correlations between the protons and carbons of the TC of *A. macrocarpa* can be established and it was verified that the structure observed for the TCs in the ^1^H and ^13^C NMR were confirmed by the HSQC analysis, being this technique known for revealing structural details of complex materials.^47^ Correlations between H/C8 referring to the resorcinol group are found centered between 5.9–6.4*/*101.9 and 104.8; in addition, correlations between H/C2′ and H/C6′ appear overlapping in the range of 5.9 and 6.4*/*108.1–111. Still referring to the aromatic ring B of flavanols, overlapping correlations between H/C2′, C5′, and C6′ appear.^47–49^ Furthermore, there is a similarity between the three HSQC measurements, indicating that there are no substantial variations when comparing tannins extracted from different regions, Figure 5.

**Figure 5:**
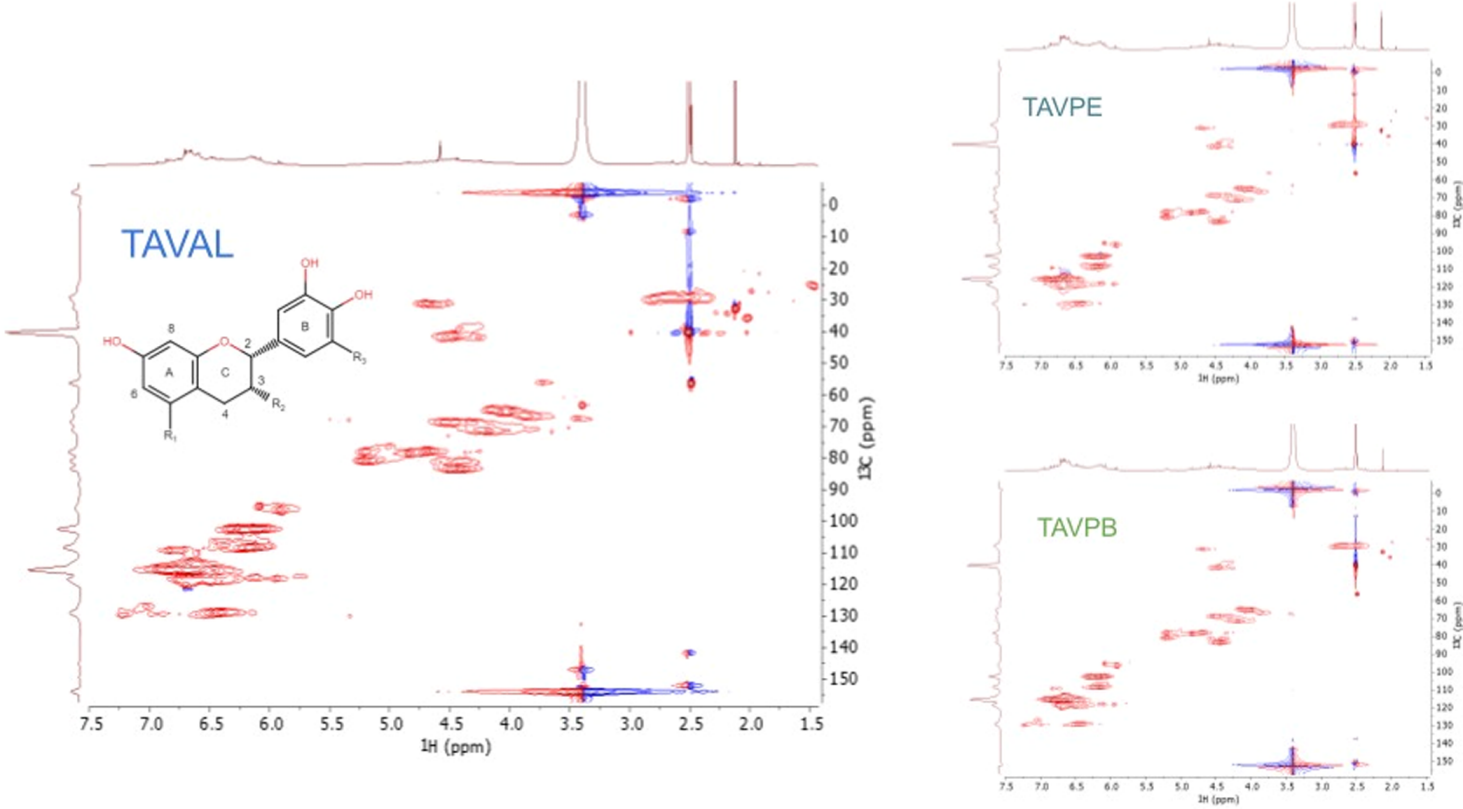
^1^H–^13^C heteronuclear single quantum coherence (HSQC) NMR spectrum of the CT of *A. macrocarpa* samples.

### 3.2 UV–Vis Spectroscopy

The UV–vis spectra of the tannin extracts from the three sampling regions (TAVPE, TAVAL, and TAVPB) exhibit a prominent absorption band centered at approximately 281 nm, Figure 6, with absorbance values presented along the *y*-axis. This band is attributed to π–π* transitions in the aromatic rings of flavanol units and is commonly associated with the A ring of catechin-type structures.^50^ It is frequently used as a spectral fingerprint of monomeric flavanols in condensed tannins.^51^ The overall spectral profiles and absorbance intensities are comparable among the samples and are consistent with previous studies on tannin-rich extracts from other plant species.^44,52^

**Figure 6:**
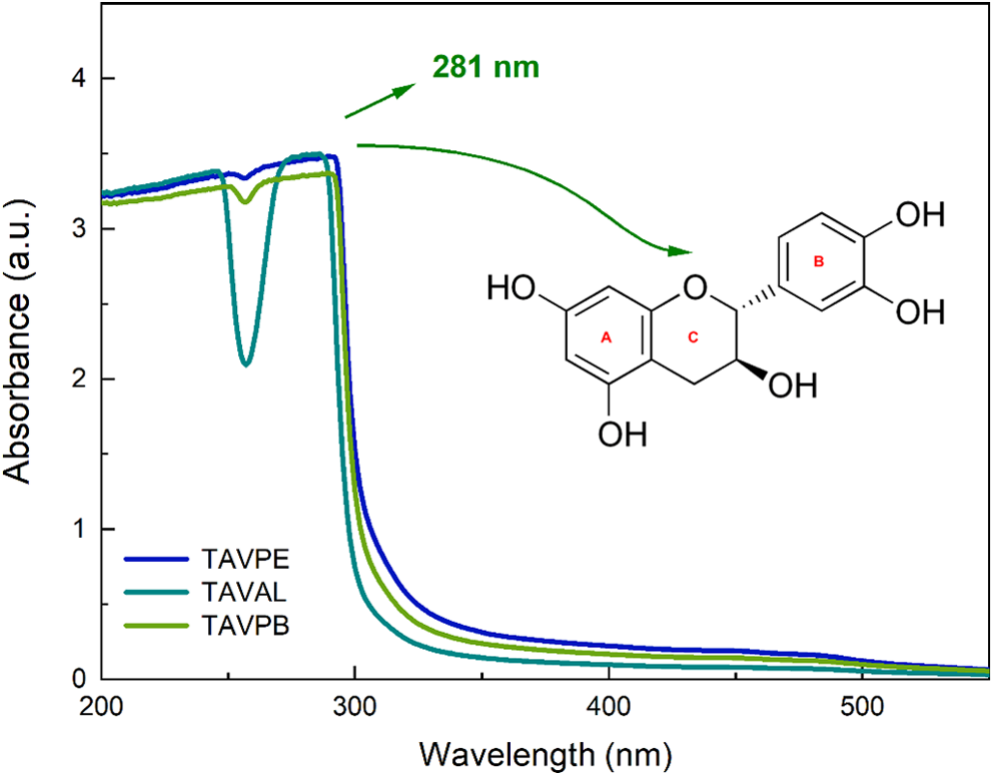
UV–vis Spectrum of *A. macrocarpa* samples.

Furthermore, the absence of significant shifts reinforces the idea of a conserved chemical environment for the chromophores involved. It is also worth noting that no shoulder peaks appeared at higher wavelengths, features that would typically indicate the presence of polymeric forms or extended conjugated systems.

### 3.3 Fourier Transform Infrared Spectroscopy (FTIR)

The tannin is composed of different types of functional groups, resulting in a complex infrared spectrum. In Figure 7, we have a comparison of the complete FTIR spectra for the different locations. It is possible to observe that in all samples, regardless of the collection site, the main absorption bands remained consistent, suggesting a conserved chemical structure. In Figure 8, we have a highlight for a strong absorption peak is found between 3500 and 3000 cm^–1^, centered at 3285 cm^–1^. This region typically provides information about the spatial arrangement and interaction between hydroxyl (OH) constituents.^53,54^ Furthermore, according to studies conducted by Ogata et al. 2005,^55^ where spectral patterns of condensed tannin were presented, peaks in the range of 3600–3000 cm^–1^ were associated with the presence of proanthocyanidins.

**Figure 7:**
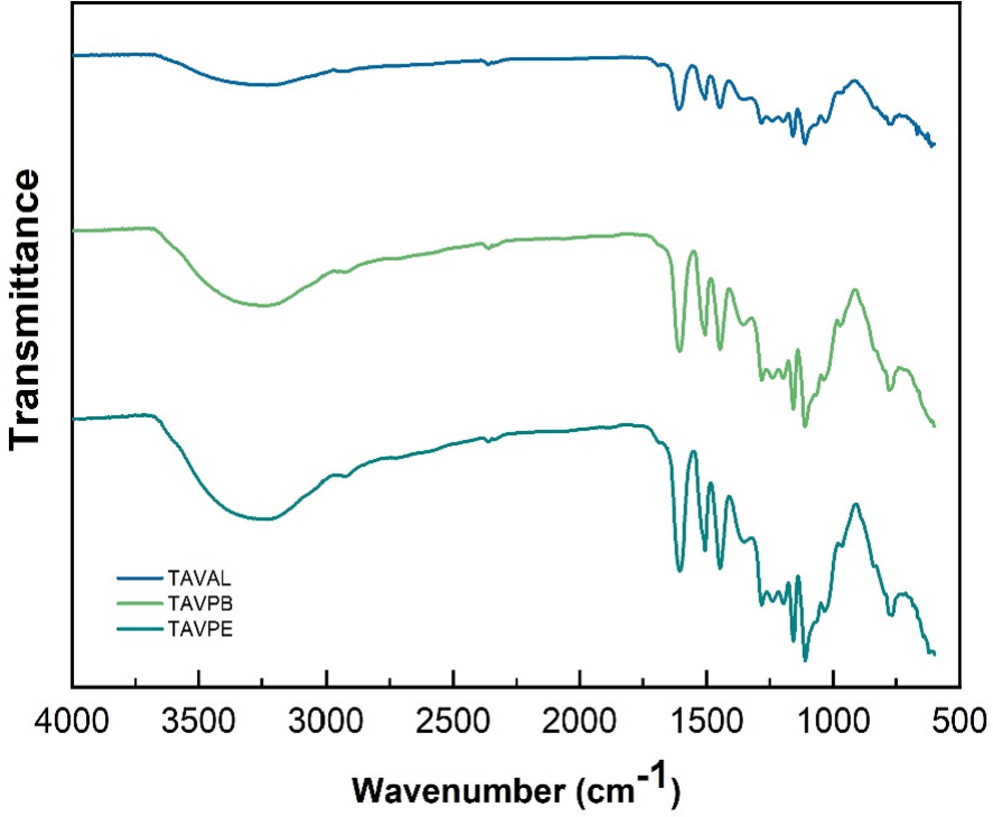
ATR-FTIR spectrum of the condensed tannin of *A. macrocarpa* samples.

**Figure 8:**
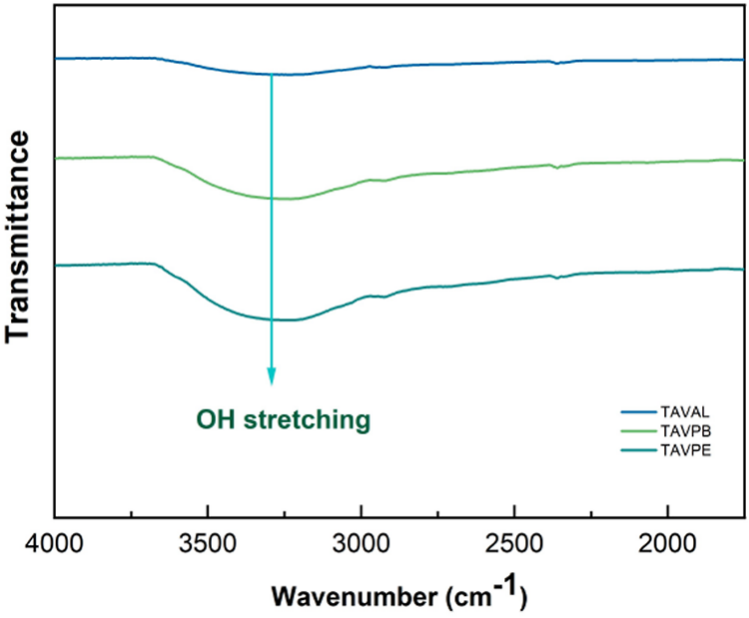
FTIR spectrum detailing the region between 4000 and 1750 cm^–1^.

The consistent presence of these hydroxyl bands across all regions suggests a conserved distribution of flavanol subunits regardless of the edaphoclimatic differences among regions. Although a variation in band intensity is observed for the TAVAL sample, such differences are not necessarily related to variations in the degree of polymerization since experimental factors inherent to the ATR–FTIR technique, including sample contact and particle size, may also influence spectral intensities.

When we started to analyze the region that would be a fingerprint of this material, we found that the peaks that appeared indicated the presence of condensed tannin, Figure 9. The peaks identified between 1600 and 1400 cm^–1^ are characteristic of aromatic compounds.^56–58^ The peak between 1288 and 1280 cm^–1^ was attributed to the asymmetric stretching vibration of the ethereal group. Additionally, peaks at 1159 (CC stretching + CH bending), 1109 (C–O stretching and O–H deformation), and 842 (C–C, C–H, C–O, and ring vibrations) are found; they are typical of condensed tannin and are consistent with procyanidin-type oligomeric structures.^18,59^ Table 3 shows the main vibrational frequencies observed in the FTIR spectrum for the purified extract of condensed tannin from *A. macroca* *rpa*.

**Figure 9:**
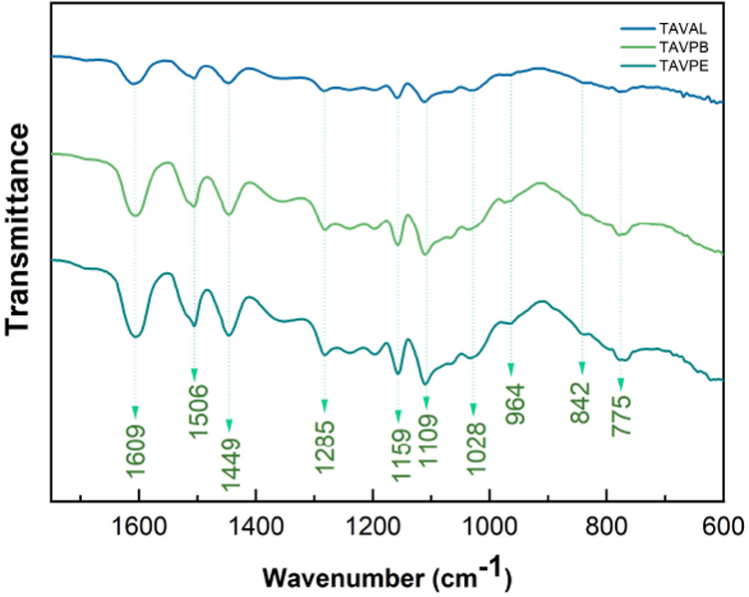
FTIR spectrum detailing the region between 1750 and 600 cm^–1^.

**Table 3:** Relationship of Peaks Found in the FTIR Spectrum of *A. macrocarpa* TC and Related Functional Groups

peak (cm^–1^)	group	refs
775	ring breathing	^60^
842	C–C, C–H, C–O, and ring vibrations	^59,61^
1028	deformation C–O–C etheric groups	^62^
1109	C–O stretching and O–H deformation	^63^
1159	stretching CC + bending CH	^59^
1285	stretching C–O ethereal structure	^59,64^
1449	aromatic ring stretching vibrations	^65^
1506	stretching C = C aromatic compounds	^66^
1609	aromatic –C = C– bondings	^61,67^
3500 – 3000	OH stretching	^18,54^

### 3.4 EPR

EPR spectroscopy has proved to be a powerful tool for identifying and studying the structure of radicals derived from phenols and, more specifically, the antioxidant potential of proanthocyanidins.^68,69^ The spectrum obtained in our studies, as illustrated in Figure 10, showed a *g*-factor of ≈2.003 for all samples. This value is characteristic of organic free radicals,^70^ capable of stabilizing unpaired electrons, a characteristic of radical scavengers, which indicates the presence of paramagnetic species.

**Figure 10:**
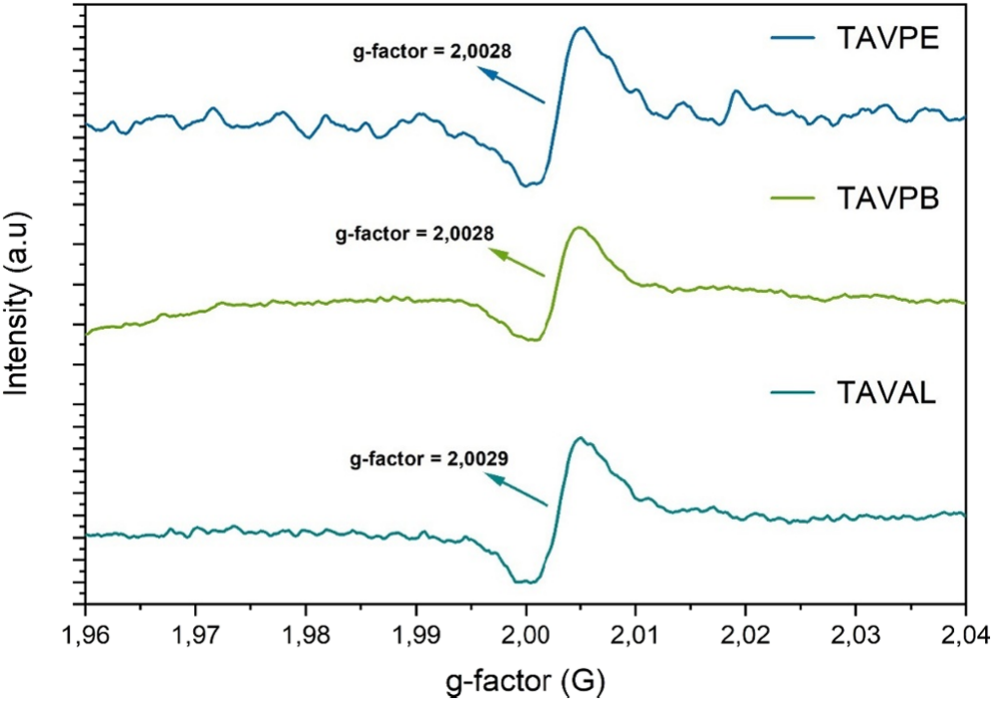
EPR plot for samples from different localities of condensed tannin from *A. macrocarpa*.

These findings are in agreement with the FTIR spectrum, which showed broad OH absorption bands between 3600 and 3000 cm^–1^, confirming the abundance of free hydroxyl groups. These species play an essential role in the antioxidant capacity of organic compounds, actively participating in reactions to neutralize reactive oxygen species. However, it is important to emphasize that not all hydroxyl groups act as strong antioxidants, even though the number of hydroxyl groups present in the substance influences its ability to scavenge free radicals;^71^ factors such as their position on the aromatic ring are fundamental in determining their function..^71–73^ Furthermore, the signals observed in the spectrum are in agreement with the EPR profiles previously described for proanthocyanidin monomers,^74^ corroborating the identification of paramagnetic species in our material.

### 3.5 Thermogravimetric Analysis (TGA)

The graph in Figure 11 is expressed in terms of mass loss as a function of temperature and represents the thermogravimetric profile of CT of *A. macrocarpa*. It is possible to observe three distinct stages of thermal degradation that correspond to specific physicochemical transitions.

**Figure 11:**
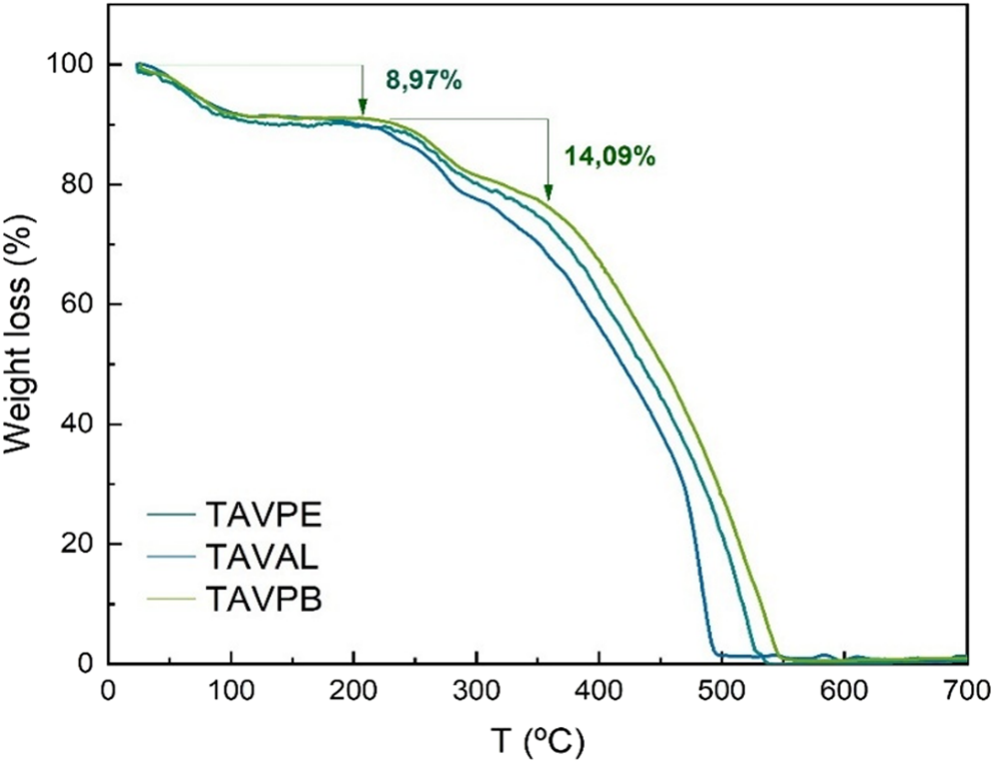
Thermogravimetric analysis of samples of *A. macrocarpa*.

The first mass loss, centered at approximately 91.2 °C, accounts for 8.97% of the total mass and is attributed to preliminary stages of oxidation and elimination of volatile fractions.^2,58,75^ The second point, where a mass loss is observed, is centered at 248^◦^ *C*, presenting a loss of approximately 14.09%. According to Pantoja-Castroa et al.^58^ this point can be considered the beginning of tannin degradation as a result of partial breakage of intermolecular bonds. At the end of this stage, a residual value of 78.09% remains. When taking the average of the three samples analyzed, it is observed that combustion occurs at 520°C, leading to complete degradation of the material and release of oxidized residues (CO_2_, CO, H_2_O).^2^

This thermal behavior, particularly the substantial residue maintained at intermediate temperatures, supports the notion of a structurally stable condensed tannin. Combined with the consistent FTIR and NMR profiles across samples from different regions, as well as their comparable antioxidant activities, the TGA results reinforce the hypothesis that *A. macrocarpa* produces chemically homogeneous and thermally stable tannins regardless of edaphoclimatic variations. This feature is particularly advantageous for large-scale extraction and industrial applications, where batch-to-batch consistency is critical.

### 3.6 Antimicrobial and Antioxidant Activity

The antimicrobial activity assessment of purified tannins from *A. macrocarpa* bark against different strains of *S. aureus* showed that the highest tested concentration (1000 μg/mL) resulted in the most effective inhibition of microbial growth, with statistically significant differences compared to the other concentrations, Table 4. This trend was observed for all samples and both bacterial strains, although the magnitude of inhibition differed between *S. aureus* and MRSA. However, for the *A. macrocarpa* sample TAVAL, the concentrations of 1000, 500, and 250 μg/mL did not show statistically significant differences against the MRSA strain, indicating a reduced sensitivity of this resistant strain to the tannin extract at these concentrations. In addition, the statistical analysis revealed that the sample TAVPB of purified tannin extracted from the bark of *A. macrocarpa* was more effective in inhibiting the growth of both strains tested in this study at most of the concentrations evaluated, suggesting that subtle differences among extracts from different collection regions may influence antimicrobial performance, particularly against MRSA.

**Table 4:** Antimicrobial Activity in % of the Purified Tannin from the Bark of *A. macrocarpa* against Species of *S. aureus* and Methicillin-Resistant *S. aureus* (MRSA)^a^

	TAVAL		TAVPE		TAVPB	
concentration (μG/ML)	*S. Aureus*	MRSA	*S. Aureus*	MRSA	*S. Aureus*	MRSA
1000	74.87 ± 1.42^aA^	34.72 ± 3.01^aC^	56.53 ± 1.76^aB^	49.63 ± 0.94^aB^	75.47 ± 0.72^aA^	71.08 ± 2.45^aA^
500	56.11 ± 1.22^bB^	31.79 ± 0.14^aB^	41.58 ± 2.21^bC^	30.63 ± 0.16^cB^	67.57 ± 2.00^bA^	60.32 ± 2.96^bA^
250	54.16 ± 0.79^bB^	34.67 ± 1.48^aB^	38.90 ± 1.90b^cC^	38.56 ± 1.16^bB^	62.43 ± 1.41^cA^	46.33 ± 1.98^cA^
125	37.86 ± 1.68^cB^	25.39 ± 1.66^bB^	33.87 ± 0.85^cdC^	30.65 ± 1.08^cB^	59.79 ± 1.18^cdA^	46.66 ± 1.79^cA^
62.5	35.14 ± 1.07^cB^	14.39 ± 1.26^cB^	29.94 ± 2.21^dB^	12.17 ± 1.36^dB^	57.18 ± 0.43^dA^	36.11 ± 0.29^dA^

a Different lowercase letters in the same column indicate a significant difference (*p* < 0.05). Different uppercase letters in the same row indicate a significant difference compared to identical microorganisms (*p* < 0.05). Results are expressed as mean ± standard deviation (*n* = 3).

Other studies have also demonstrated the antimicrobial potential of condensed tannins extracted from different plant species against a variety of bacterial strains.^76–79^ For example, studies conducted by Shi et al.^80^ evaluated the antimicrobial activity of condensed tannins extracted from rice straw against *S. aureus*, showing that the material exhibited a strong inhibitory effect. Moreover, the study found that these tannins were also capable of regulating the biofilm morphology. Furthermore, García-Rodriguéz et al.^81^ working with condensed tannins from coffee pulp (*Coffea arabica*), observed inhibition zones against *S. aureus* only at concentrations of 5 and 10 g/L. And Jian et al.^82^ evaluating condensed tannins from *Cercis chinensis* Bunge leaves, observed MIC for *S. aureus* at a concentration of 1.5 mg/mL.

The inhibitory mechanisms of condensed tannins were evaluated in a study by Huang et al.^83^ using *Pediococcus pentosaceus*, a Gram-positive bacterium. The study observed that the inhibition is related to the suppression of protease activity and may also be partially associated with oxidative stress, reinforcing that condensed tannins can exert antibacterial effects through multiple interaction pathways.

Despite the overall similarity of the chemical profiles, significant variation was observed when the same dosage was used for samples from different regions. In particular, there was a greater decrease in the antimicrobial activity against the MRSA strain for the TAVAL sample. According to some studies on phenolic compounds, including tannins and related polyphenols, the antimicrobial effects against MRSA may involve interactions with the bacterial cell membrane, leading to alterations in membrane fluidity and integrity, inhibition of efflux pumps, and inhibition of β-lactamases.^84,85^ For example, studies conducted by Chew et al.^85^ showed that in bioactive extracts against MRSA, membrane disruption and leakage of cellular contents were observed as key elements of the antibacterial action of tannin-rich extracts, indicating that subtle structural differences can modulate these effects. These interactions vary depending on the chemical structure of the phenolic compounds and the intrinsic tolerance mechanisms of MRSA, which may explain the differential inhibition observed between samples with similar overall chemical profiles.

It is important to note that the antimicrobial and antioxidant activities of tannins are interconnected due to common molecular mechanisms. For example, the chelation of metal ions, which provides an antioxidant action, also favors the inhibition of extracellular microbial enzymes or the deprivation of substrates essential for bacterial growth, mechanisms that explain the antimicrobial activity of tannins.^72,73,86^ Therefore, an examination of their antioxidant performance is warranted as it provides complementary insight into the molecular determinants that may also contribute to the observed antimicrobial profiles.

Thus, the antioxidant potential of the condensed tannin extracts from *A. macrocarpa* was assessed using four complementary assays: ABTS and DPPH radical scavenging and copper and iron chelating activity. For each assay, different concentrations were tested—1000, 500, 250, 125, and 62.5—statistical comparisons were performed between the extracts obtained from the three collection regions: TAVPB, TAVPE, and TAVAL.

Overall, the samples showed a moderate antioxidant activity. Regarding ABTS scavenging activity, the 1000 μg/mL concentration presented the best results, Table 5. It is important to highlight that, for all concentrations evaluated, there was a significant difference (*p* < 0.05) between samples collected from different locations. The sample that demonstrated the best performance in this assay was TAVPE, precisely at the 1000 μg/mL concentration.

**Table 5:** Antioxidant Activity in μmol of Trolox Equivalent (TE) for the Scavenging of ABTS Radicals of the Tannin Purified from the Bark of *A. macrocarpa* ^a^

concentration (μG/Ml)	TAVPB	TAVPE	TAVAL
1000	713.90 ± 12.14^aB^	764.38 ± 8.27^aA^	679.14 ± 8.41^aC^
500	662.47 ± 15.66^bB^	755.81 ± 6.42^aA^	603.90 ± 9.35^bC^
250	614.38 ± 6.42^cB^	710.09 ± 7.03^bA^	531.52 ± 12.84^cC^
125	527.71 ± 13.99^dB^	652.47 ± 11.50^cA^	427.71 ± 6.06^dC^
62.5	378.66 ± 4.85^eB^	611.52 ± 4.09^dA^	277.24 ± 15.49^eC^

a Different lowercase letters in the same column indicate a significant difference (*p* < 0.05). Different uppercase letters in the same row indicate a significant difference (*p* < 0.05). Results are expressed as mean ± standard deviation (*n* = 3).

As shown in Table 6, all samples demonstrated concentration-dependent antioxidant activity in the DPPH assay. The maximum activity was observed at the highest concentration tested, with extracts from all three regions showing statistically similar scavenging efficiencies (*p* > 0.05). This suggests the DPPH radical scavenging capacity of *A. macrocarpa* tannins is preserved regardless of the edaphoclimatic conditions of the collection site. The values obtained are comparable to those reported for other condensed tannin-rich plant extracts and are consistent with the presence of phenolic hydroxyl groups identified by FTIR and NMR analyses.

**Table 6:** Antioxidant Activity in μmol of Ascorbic Acid Equivalent (AAE) for the Scavenging of DPPH Radicals of the Tannin Purified from the Bark of *A. macrocarpa* ^a^

concentration (μG/ML)	TAVPB	TAVPE	TAVAL
1000	22.09 ± 0.19^aA^	23.00 ± 0.41^aA^	22.32 ± 0.51^aA^
500	22.35 ± 0.19^aB^	23.10 ± 0.22^aA^	21.59 ± 0.27^aC^
250	19.81 ± 0.44^bB^	22.32 ± 0.16^abA^	15.65 ± 0.44^bC^
125	17.54 ± 0.64^cB^	20.94 ± 0.32^bA^	10.06 ± 0.31^cC^
62.5	15.96 ± 0.30^dA^	16.65 ± 1.27^cA^	5.62 ± 1.13^dB^

a Different lowercase letters in the same column indicate a significant difference (*p* < 0.05). Different uppercase letters in the same row indicate a significant difference (*p* < 0.05). Results are expressed as mean ± standard deviation (*n* = 3).

The metal complexing capacity of purified tannins from *A. macrocarpa* bark was evaluated against Fe^2+^ and Cu^2+^ ions, revealing interesting and consistent behavior among the samples (Tables 7 and 8). In general, the extracts showed greater chelating activity for iron than for copper, especially at the highest concentrations tested. In the case of iron, the three samples exhibited high complexation percentages, exceeding 50% at concentrations of 1000, 500, and 250 μg/mL, with no statistically significant differences among them, suggesting good stability of the chelating activity regardless of the collection region. In contrast, for cupric ions, greater variability was observed among the samples, with TAVPE presenting the best results at almost all concentrations tested, particularly at 1000 μg/mL, at which it achieved approximately 40% chelating activity. These results indicate that, although *A. macrocarpa* tannins are efficient in complexing metal ions, especially iron, environmental factors may more strongly influence the interaction with copper.

**Table 7:** Copper Chelating Activity in % of the Tannin Purified from the Bark of *A. macrocarpa* ^a^

CONCENTRATION (μG/ML)	TAVPB	TAVPE	TAVAL
1000	34.73 ± 0.74^aB^	39.70 ± 2.69^aA^	39.32 ± 1.70^aA^
500	31.97 ± 0.58^aC^	38.20 ± 0.98^aA^	35.20 ± 1.06^bB^
250	24.71 ± 2.62^bC^	38.76 ± 1.75^aA^	31.64 ± 1.04^cB^
125	18.54 ± 0.59^cC^	31.46 ± 0.39^bA^	28.27 ± 0.97^dB^
62.5	16.34 ± 0.43^cB^	25.65 ± 0.65^cA^	25.70 ± 0.66^dA^

a Different lowercase letters in the same column indicate a significant difference (*p* < 0.05). Different uppercase letters in the same row indicate a significant difference (*p* < 0.05). Results are expressed as mean ± standard deviation (*n* = 3).

**Table 8:** Iron Chelating Activity in % of the Tannin Purified from the Bark of *A. macrocarpa* ^a^

concentration (μG/ML)	TAVPB	TAVPE	TAVAL
1000	55.36 ± 0.55^aA^	56.15 ± 1.01^aA^	55.58 ± 1.04^aA^
500	55.13 ± 0.89^aA^	53.21 ± 0.96^aA^	53.54 ± 0.77^aA^
250	52.31 ± 1.99^aA^	55.98 ± 2.13^aA^	54.79 ± 1.90^aA^
125	46.38 ± 0.21^bB^	54.23 ± 163^aA^	4706 ± 2.52^bB^
62.5	22.91 ± 1.33^cC^	48.92 ± 0.89^bA^	37.64 ± 2.81^cB^

a Different lowercase letters in the same column indicate a significant difference (*p* < 0.05). Different uppercase letters in the same row indicate a significant difference (*p* < 0.05). Results are expressed as mean ± standard deviation (*n* = 3).

According to Zeng et al., condensed tannins are recognized for their effectiveness in chelating metal ions.^87^ This property is primarily attributed to the presence of galloyl or catechol groups located in the B ring of their flavan-3-ol units. Moreover, the interaction between condensed tannins and metal ions occurs through two main mechanisms: ion exchange and complexation.

Although the three samples exhibit overall chemical similarity, as supported by their FTIR and NMR profiles, subtle variations within the tannin structures can influence their biological and antioxidant responses.^88,89^ Condensed tannins are structurally heterogeneous materials whose functional performance depends not only on the presence of specific phenolic moieties but also on their relative abundance, degree of polymerization, and stereochemistry. These fine-scale differences may not significantly alter the global similarity metrics applied here; however, they are sufficient to modulate mechanisms such as radical scavenging, metal chelation, and interactions with biological targets.^72,86^ Therefore, the distinct antioxidant and biological outcomes observed among the samples do not contradict their chemical resemblance; rather, they reflect a functional sensitivity to subtle but relevant structural variations within the tannin framework.

## 4 Conclusions

The study demonstrated that condensed tannins extracted from the bark of *A. macrocarpa* exhibit a high degree of chemical consistency across different collection regions, with no detectable differences within the sensitivity limits of the spectroscopic techniques employed, regardless of environmental variations. Spectroscopic analyses confirmed the absence of hydrolyzable tannins and revealed well-defined phenolic structures whose stability is directly associated with the observed biological efficacy. Antioxidant and antimicrobial activities, including efficacy against resistant strains, reinforce the bioactive potential and the reliability of the material. The extraction process yielded 125.7 g of condensed tannins per kilogram of dry bark, reinforcing the robustness and scalability of the raw material supply.

Although the samples display highly similar chemical profiles, subtle structural nuances inherent to natural matrices may contribute to the statistically detectable variations observed in antioxidant and antimicrobial assays. These variations, however, do not compromise the overall functional consistency of the material and remain within the range expected for plant-derived polyphenols, thereby preserving its reliability for practical use. Thus, condensed tannins from *A. macrocarpa* represent a standardizable and promising resource for large-scale industrial applications.

## Supplementary Material



## Notes

^13^C NMR spectra (180–0 ppm) and ^1^H NMR spectra (10–0 ppm) of the condensed tannin samples from each collection site, provided separately for all analyzed samples (PDF)
