# Supplementary material for: Chemical and Functional Consistency of Condensed Tannins from Anadenanthera macrocarpa at Different Collection Sites
Source: ACS Omega. 2026 Feb 17;11(8):13199–211. doi: 10.1021/acsomega.5c09054 (PMC12961482; doi:10.1021/acsomega.5c09054)
Supplement: Supplementary file 1 [file ao5c09054_si_001.pdf]

Chemical and functional consistency of condensed tannins from *Anadenanthera macrocarpa* at different collection sites

Kamilla V R de A Silva[1,*] , José Erick Galindo Gomes[2], Keila Aparecida Moreira[2], Rodrigo José da Silva Lima[3,4], Pedro L.Guzzo[5], Dulciene Karla de Andrade Silva[2], E. Padrón-Hernández [6,7]

1.Universidade Federal de Campina Grande, Unidade Acadêmica de física, Rua Aprígio Veloso, 58109-970, Campina Grande, PB, Brazil.

2.Universidade Federal do Agreste de Pernambuco, Laboratório de Microbiologia, Tecnologia Enzimática e Bioprodutos, Av. Bom Pastor, s/n, 55292-270, Garanhuns, PE, Brazil.

3.Universidade Federal de Campina Grande, Laboratório de Espectroscopia Fotoacústica, Rua Aprígio Veloso, 58109-970, Campina Grande, PB, Brazil.

4.University of Copenhagen, Departament of Chemistry, Universitetsparken 5, 2100, Copenhagen Ø, Denmark.

5.Universidade Federal de Pernambuco, Departamento de Engenharia de Minas, Av. Prof. Moraes Rego, 1235, 50670-901 Recife, PE, Brazil.

6.Universidade Federal de Pernambuco, Departamento de Física, Av. Jorn. Aníbal Fernandes, s/n, 50740-540, Recife, PE, Brazil.

7.Universidade Federal de Pernambuco, Pós-graduação em Ciência dos Materiais, Av. Jorn. Aníbal Fernandes, s/n, 50740-540, Recife, PE, Brazil.

*Corresponding author: kamilla.rodrigues@df.ufcg.edu.br

Supporting Information

- ^{13}C RMN

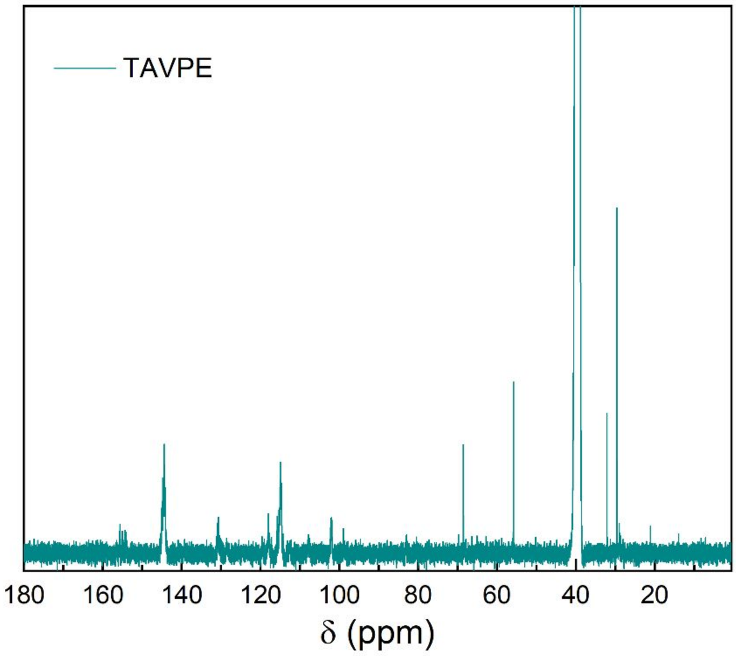

Figure S1: ^{13}C NMR spectrum of condensed tannin from *A. macrocarpa* in DMSO-d6 from TAVPE sample. .

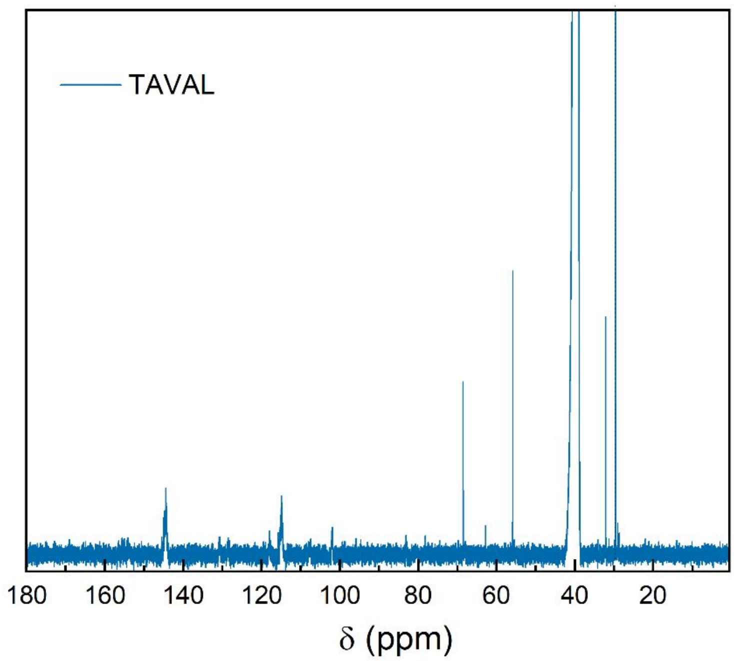

Figure S2: ^{13}C NMR spectrum of condensed tannin from *A. macrocarpa* in DMSO-d6 from TAVAL sample.

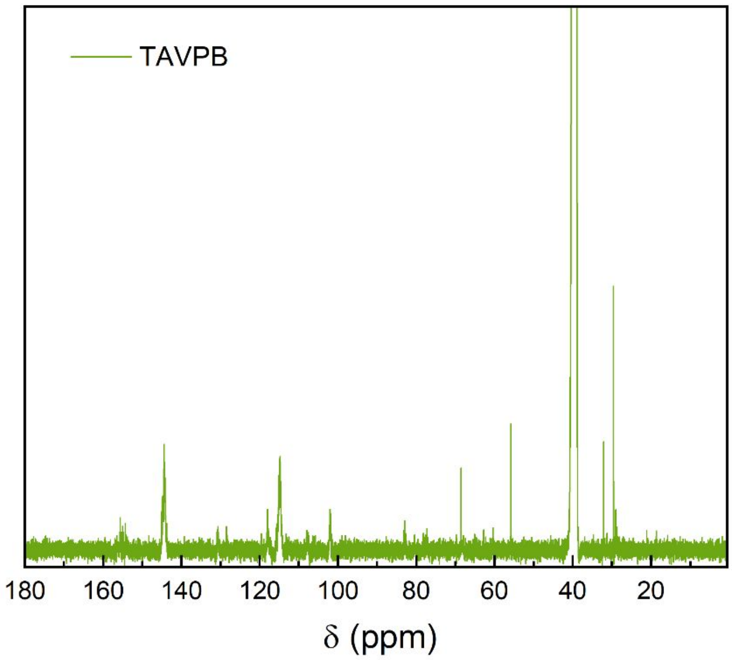

Figure S3: ^{13}C NMR spectrum of condensed tannin from *A. macrocarpa* in DMSO-d6 from TAVPB sample.

- ^{1}H RMN

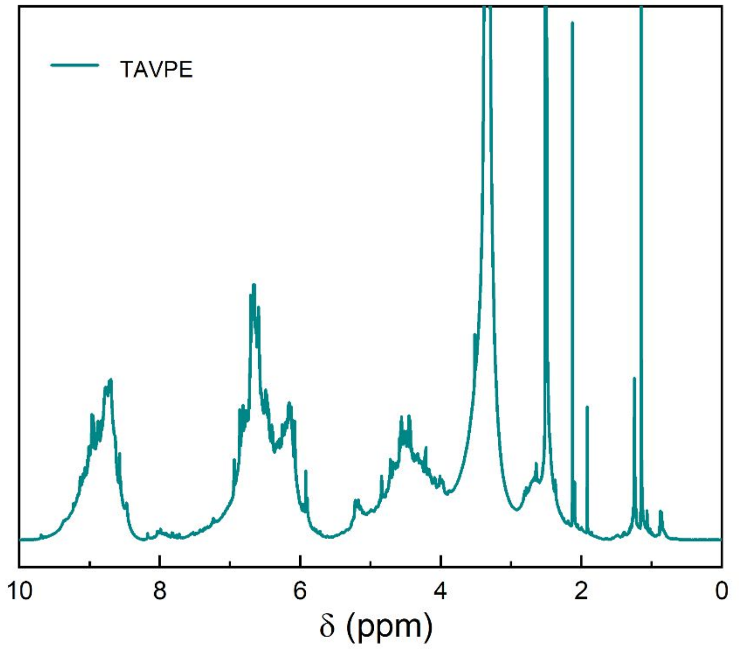

Figure S4: ^{1}H NMR spectrum of condensed tannin from *A. macrocarpa* in DMSO-d6 from TAVPE sample.

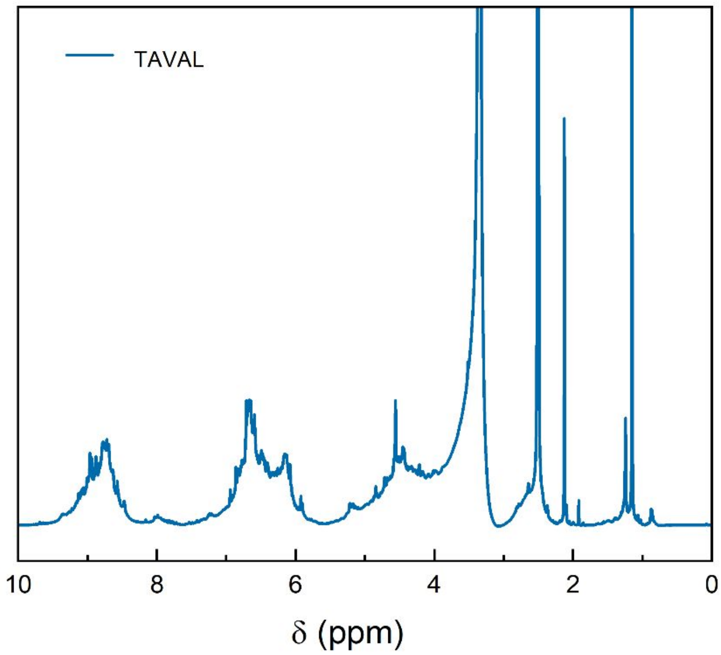

Figure S5: ^{1}H NMR spectrum of condensed tannin from *A. macrocarpa* in DMSO-d6 from TAVAL sample.

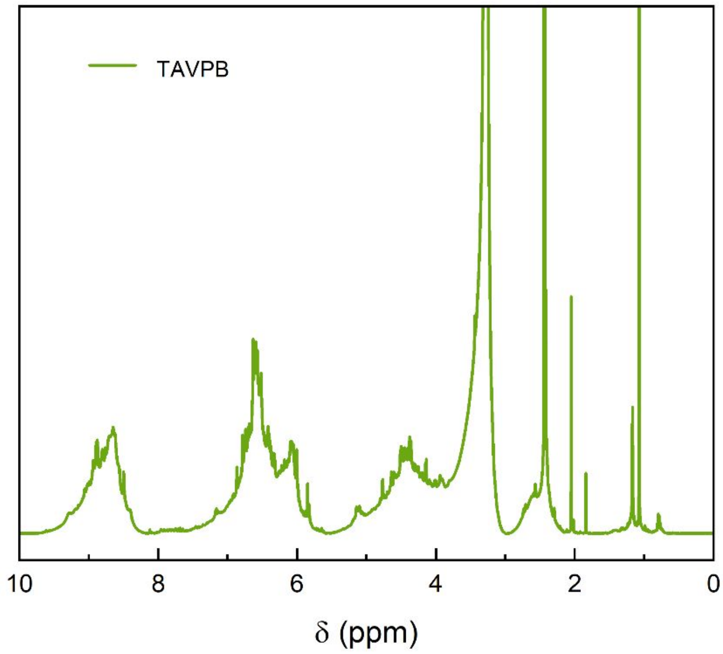

Figure S6: ^{1}H NMR spectrum of condensed tannin from *A. macrocarpa* in DMSO-d6 from TAVPB sample.
